# Ultrathin organic membranes: Can they sustain the quest for mechanically robust device applications?

**DOI:** 10.1016/j.isci.2023.105924

**Published:** 2023-01-05

**Authors:** Elena Missale, Marco Frasconi, Maria F. Pantano

**Affiliations:** 1 Department of Civil, Environmental and Mechanical Engineering, University of Trento, Via Mesiano 77, 38123 Trento, Italy; 2 Department of Chemical Sciences, University of Padova, Via Marzolo 1, 35131 Padova, Italy

**Keywords:** Organic chemistry, Membranes, Engineering

## Abstract

Ultrathin polymeric films have recently attracted tremendous interest as functional components of coatings, separation membranes, and sensors, with applications spanning from environment-related processes to soft robotics and wearable devices. In order to support the development of robust devices with advanced performances, it is necessary to achieve a deep comprehension of the mechanical properties of ultrathin polymeric films, which can be significantly affected by confinement effects at the nanoscale. In this review paper, we collect the most recent advances in the development of ultrathin organic membranes with emphasis on the relationship between their structure and mechanical properties. We provide the reader with a critical overview of the main approaches for the preparation of ultrathin polymeric films, the methodologies for the investigation of their mechanical properties, and models to understand the primary effects that impact their mechanical response, followed by a discussion on the current trends for designing mechanically robust organic membranes.

## Graphical abstract

**Figure:**
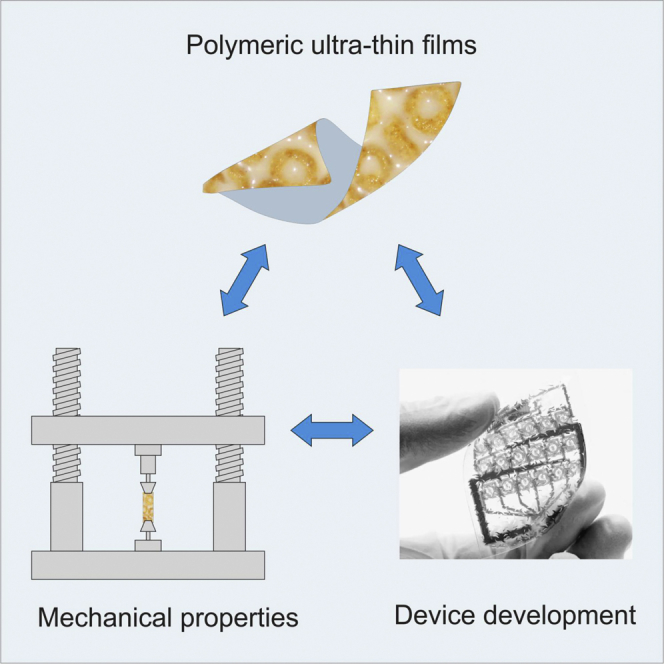

## Introduction

**Figure 1:**
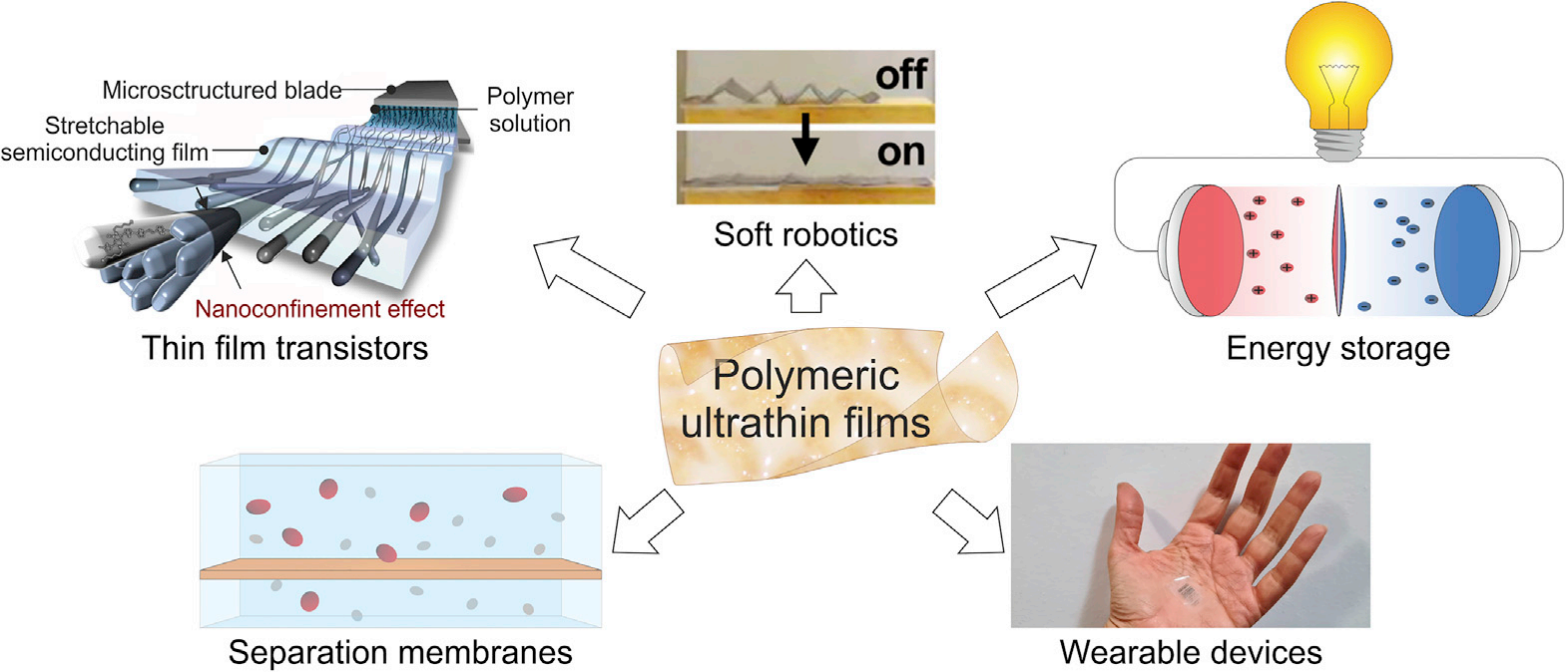
Ultrathin polymeric films applications Overview of applications of ultrathin films, such as thin-film transistors (reproduced with permission from Wu et al.^5^), soft robotics (reproduced with permission from Schmauch et al.^9^), energy storage, wearable devices, and separation membranes.

Polymeric films, especially with nanoscale thickness, are attracting a tremendous interest due to perspective applications spanning a variety of fields, from environment and energy-related separation/transportation to soft robotics (Figure 1). Ultrathin polymeric films represent ideal functional components of the next-generation membranes for gas separation,^1^ desalinization^2^ and nanofiltration,^3^ thin-film transistors,^4^^,^^5^ wearable sensing devices,^6^ long-life high-capacity batteries,^7^ and miniaturized soft robots,^8^^,^^9^^,^^10^ whose efficiency and performance can in principle greatly benefit from thickness reduction down to the nanoscale.

Stiff glassy and soft viscoelastic polymers have been widely used for the fabrication of ultrathin membranes for filtration^11^ and as protective coatings.^12^ In the last decade, the progress in flexible and wearable technologies has accelerated the development of conductive and semiconductive polymeric films with thickness from a few μm to tens of nanometers.^4^^,^^13^ On the other hand, 2D polymeric films, which are covalently linked networks of monomers with periodic bonding,^14^ have emerged as disruptive nanoporous materials for applications such as gas-selective membranes^15^^,^^16^ and osmotic power generators.^17^ Among them, 2D covalent organic frameworks (COFs) have attracted particular interest owing to their highly tunable electronic and molecular transport properties and their thermal and chemical stability.^18^^,^^19^

While different strategies have been devised to fabricate polymeric films with controlled thickness and well-defined electronic and transport properties,^20^^,^^21^ the design of high-performance yet reliable and robust devices comprising polymeric films has to consider their eventual exposure to various kinds of stress, as a consequence of traction, compression, bending, or torsion loads acting in real operating conditions and, thus, has to be underpinned by a deep comprehension of the mechanical behavior of all components at the specific length scale involved. It has been shown that mechanical properties, such as Young’s modulus and strength, may deviate even significantly from their bulk values, when materials are confined to nanometric size, as for ultrathin films.^22^^,^^23^^,^^24^ While size effects are now well known in thin metal films or wires,^25^^,^^26^ in the case of polymeric films, there still exist ambiguities and controversies, as evident, for example, from the conflicting dependence of Young’s modulus on film’s thickness that has been observed and reported even for common materials.^27^ In order to shed light on this matter, extensive investigations would be necessary, but these are made quite challenging by the small size and peculiar aspect ratio of the samples to be tested, which require conventional macroscopic testing equipment to be replaced by *ad-hoc* experimental strategies. In this review paper, we aim at discussing the main experimental approaches that have been proposed for the mechanical characterization of ultrathin polymeric films, pointing out advantages and limitations in order to provide the reader with a critical overview. Challenges related to the preparation and transfer of nanometer-thin films for the mechanical characterization are also discussed. Following that, the main mechanical properties, namely Young’s modulus, strength, and strain at failure, of selected ultrathin polymers are reviewed. We will then conclude with a glance at the perspectives and emerging trends in the field.

## Preparation and transfer of ultrathin polymeric films

**Figure 2:**
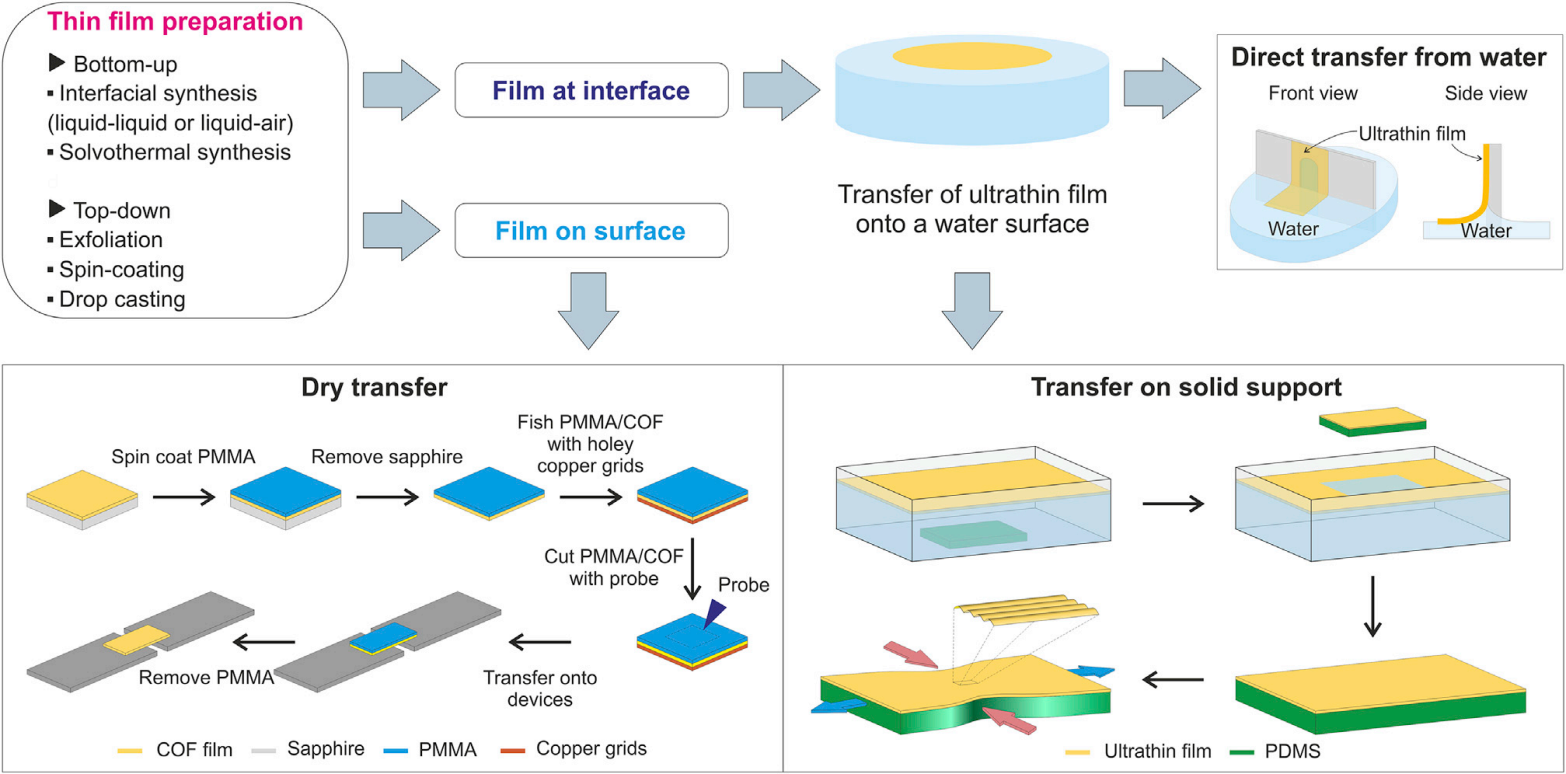
Thin film preparation and transfer Fabrication of ultrathin polymeric films and schematic of the processes for transferring thin films to different substrates for mechanical testing by using direct transfer from water (for films at interface) or dry transfer methods (for films on a solid substrate).

Polymeric thin films can be obtained as freestanding films or supported on substrates by both bottom-up and top-down strategies (Figure 2). In general, the deposition of polymeric films onto different substrates can be easily achieved by spin coating^28^ or drop casting a polymer solution or dispersion in a variety of solvents.^13^^,^^29^ Uniform ultrathin films of glassy polymers, such as poly(methylmethacrylate) (PMMA)^30^ and polystyrene (PS),^31^ and conductive polymers based on poly(3,4-ethylenedioxythiophene)/poly (styrenesulfonate) (PEDOT/PSS)^32^ with thickness from few nanometers to hundreds of nanometers have been fabricated by spin coating. The spin coating method allows for a rapid fabrication of large-area uniform films on different substrates, although the solvent employed for the preparation of the polymer solution can have a strong impact on the structure and morphology of the thin film. Indeed, fast solvent evaporation rate during the spin coating process can lead to relatively poor molecular ordering of the polymer chains.^33^^,^^34^ Slow evaporation, as it occurs in drop casting methods, can result in molecularly ordered polymeric structures with conductive, transmittance, and mechanical properties suitable for flexible electronics applications. For example, Lee et al. demonstrated the preparation of polymeric films of polyaniline doped with camphorsulfonic acid (PANI:CSA) by drop casting that yielded a higher conductivity and transmittance compared to the conventional spin-cast films.^29^ In this case, drop casting method leads to a better alignment of the polymer chains as confirmed by spectroscopic and morphological studies of the prepared films.

For 2D polymeric films, such as 2D COFs, top-down strategies involving mechanical delamination and chemical or solvent-assisted exfoliation have been employed for the preparation of single- or few-layered COF thin films from bulk materials.^35^ On the other hand, bottom-up strategies, which include solvothermal synthesis, room temperature vapor-assisted conversion, and interfacial polymerization, have enabled the preparation of 2D polymeric films with controllable thickness and surface properties.^20^ In particular, interfacial polymerization approaches, which involved the growth of the film in a confined interface region, i.e. liquid/liquid interface or liquid/air interface,^36^ have been widely used for the preparation of freestanding nanofilms based on 2D COFs^37^^,^^38^^,^^39^ and other polymeric materials, such as polyimine^3^ and polyimide^11^ nanofilms. For example, a smooth freestanding polyamide membrane with a thickness of 8 nm has been prepared by polymerization at an aqueous-organic interface.^11^ Indeed, the thickness of the films prepared by interfacial polymerization can be tuned by the monomer concentration, and the films generated at the interface are easily transferrable to arbitrary substrates for further characterizations and applications. For further details on the methods for the synthesis of ultrathin polymer films and 2D COF films, the reader is referred to the excellent reviews.^20^^,^^35^^,^^36^

Clearly, the synthetic strategy employed for the fabrication of polymeric thin films plays a substantial role in their properties and correlates with the mechanical characterization approach selected to derive the mechanical properties. Because the majority of synthetic approaches produce films floating on water or deposited on a solid supporting substrate, a critical step is the transfer of the thin film to the stage for the mechanical test without damaging the film. The primary method to prepare polymeric ultrathin film suitable for mechanical characterization tests involves the direct transfer of the film from the water surface to the specific substrate. For example, Bay & Crosby prepared high-quality freestanding PS films, as thin as 30 nm, on a slotted frame to perform tensile tests.^40^ The films floating on water were picked up perpendicularly by the frame with the open end facing down in order to prevent the film from collapsing. The film adhering to all three sides of the frame is then laser cut in “dog-bone” shape.

Transfer of ultrathin films by using removable substrates has been developed in order to avoid additional stress on the transferred film, as instead is observed by using peeling transfer methods. Ruoff and co-workers have developed an efficient method to transfer large-area films with thickness of 100 nm by using camphor as a sacrificial substrate. First, the film supported onto a layer of camphor was transferred onto hollow substrates; camphor was then sublimated, leaving a freestanding film without deformation.^41^ Another procedure called SMART (shear motion-assisted robust transfer) was recently developed for fabricating freestanding PS films with thickness down to 19 nm for mechanical testing.^31^

Briefly, a “dog-bone”-shaped PS film supported by a silicon substrate with a sublayer of water-soluble poly(sodium 4-styrenesulfonate) (PSS) was attached at both ends to two polydimethylsiloxane (PDMS)-coated grips. Upon dissolution of the PSS layer, a freestanding PS film on water is obtained with limited distortion to thin-film samples. For the mechanical characterization of films on solid substrates, films floated on water are often transferred directly onto an elastomeric support of PDMS.^42^ For the preparation of the PDMS-supported film, special care has to be taken on the adhesion of the film to the elastomer, which is achieved by a drying process. In order not to induce possible damage to thin films floating on water, a dry transfer method was developed for COF films and other 2D materials.^43^^,^^44^ This approach involved the deposition, by spin coating, of a thin sacrificial layer of PMMA on the 2D COF film supported by a solid substrate, followed by cutting by a tungsten tip and transferring the PMMA attached to the COF film onto the testing device. After annealing under H_2_ for removing the PMMA layer, the COF film is ready for the test.

## Methodologies for the mechanical characterization of ultrathin polymeric films

**Figure 3:**
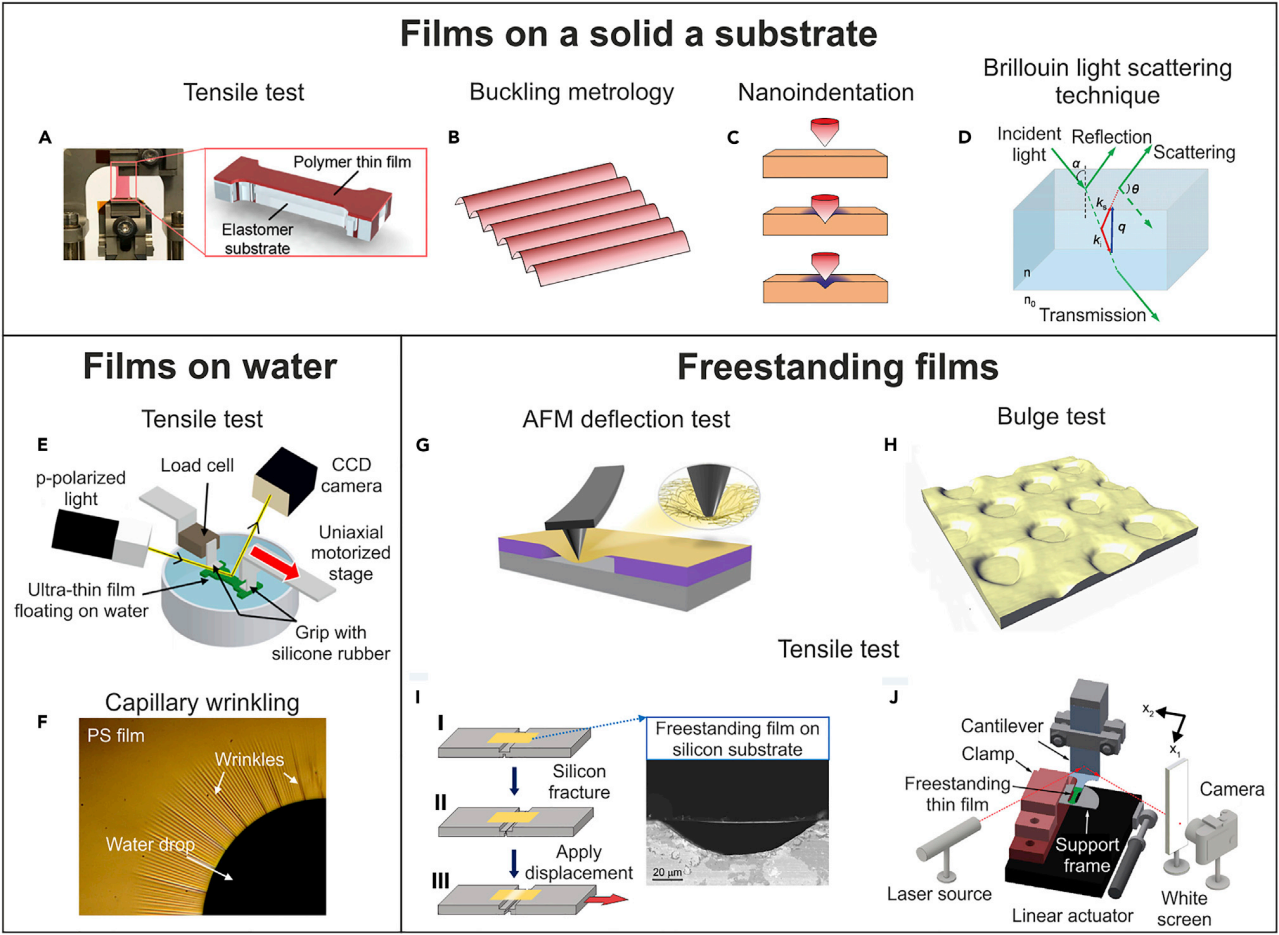
Mechanical characterization of ultrathin polymer films Testing strategies involving films supported by a solid substrate: (A) tensile test (adapted with permission from Song et al.^45^), (B) buckling metrology, (C) nanoindentation, and (D) Brillouin light scattering (reproduced with permission from Gomopoulos et al.^47^). Testing strategies involving a film supported by a water layer: (E) tensile test (reproduced with permission from Saito et al.^48^) and (F) capillary wrinkling (reproduced with permission from Chang et al.^49^). Testing strategies involving freestanding films: (G) AFM deflection test (reproduced with permission from Wang et al.^30^), (H) bulge test (reproduced with permission from O’Connell and McKenna^50^), and (I, J) tensile tests (reproduced with permission from Pantano et al.^38^ and, Bay and Crosby,^40^ respectively).

The peculiar aspect ratio of ultrathin films causes their manipulation, gripping, and testing to face several challenges, especially when the film thickness becomes smaller than 200 nm.^45^ During the last couple of decades, such challenges have been addressed via different routes, including, for example, the performance of tests on ultrathin films supported by a substrate. However, as it will be discussed in the following section, while the presence of a substrate may facilitate the preparation of a film with nanoscale thickness, it may influence the observed mechanical response, thus requiring complex data processing.^46^ We can classify the diverse experimental techniques for the mechanical characterization of ultrathin polymeric films in three categories according to the specimen testing condition (Figure 3): (i) tests on specimens supported by a solid substrate, (ii) tests on specimens supported by a liquid film, and (iii) tests on specimens that are completely freestanding.

### Mechanical testing of film supported by solid substrate

One approach to the mechanical characterization of ultrathin polymeric films relies on the preparation of laminated samples, which consist of the thin film under investigation deposited onto an elastomeric support with a few micrometers thickness.^45^ Using this strategy, the sample can be easily handled, clamped, and stretched by a universal tensile testing machine; nevertheless, the thin film has to adhere strongly to the substrate, otherwise delamination may occur, thus compromising the test success. With this approach, apart from quasi-static tests, it is possible to perform cyclic strain measurements by exploiting the force released by the elastomeric support during the unloading phase.^45^ As an alternative to tensile tests, other testing configurations can be considered, including both contact techniques, such as nanoindentation, and noncontact techniques, such as buckling metrology or Brillouin light scattering (BLS). Nanoindentation is a powerful technique largely used to derive the Young’s modulus, hardness, and fracture toughness of a variety of ceramics and metals,^51^ yet it is frequently applied also to polymeric thin films. It relies on the force-displacement curve and footprint/cracks accompanying the penetration of a tip with standard geometry within the sample under testing. In the case of thin films, additional caution is required, in order to take into account possible artifacts due to the impact with the tip and the presence of the substrate underneath the film. Indeed, the measured mechanical response can be affected by the properties of the stiffer substrate to an even significant extent (substrate effect).^52^ In the case of buckling metrology, the thin film to be tested is placed onto a pre-stretched thick elastomeric support; upon strain release in the elastomeric substrate, buckling instabilities occur that cause the appearance of a wrinkle pattern onto the film. From the analysis of such wrinkle pattern, it is possible to derive the film’s Young’s modulus, *E*_*f*_, as follows^53^^,^^54^^,^^55^^,^^56^^,^^57^:

(Equation 1) $$E_{f}=\frac{\left(1-v_{f}^{2}\right)}{\left(1-v_{s}^{2}\right)}{3E}_{s}{\left(\frac{\lambda }{2\pi h}\right)}^{3}$$

where $v_{f}$ and $v_{s}$ are the Poisson’s ratio of the film and substrate, respectively; $E_{s}$, the elastic modulus of the substrate; h, the thickness of the film; and λ, the critical wavelength characterizing the wrinkle pattern, which can be evaluated, for example, using a small-angle light scattering technique. The effectiveness of Equation 1 is underpinned by a number of assumptions, including a significant difference between the Young’s modulus of the film and that of the substrate (which has to be much thicker), strong adhesion between the film and the substrate in order to avoid slippage, shear forces are neglected, and all deformations are considered elastic.^58^

BLS is a noncontact technique, which allows us to derive the Young’s modulus of a supported thin film from the analysis of the inelastically scattered light when that is excited by a laser beam.^47^ According to the geometry backscattering setup, the elastic modulus of the film can be derived both in-plane and perpendicular to the surface.^47^

### Mechanical testing of film on liquid

Owing to the high surface tension of water, small objects, like ultrathin films, remain afloat and can slide almost frictionlessly on its surface.^59^ Such a property has been exploited in water-assisted tensile tests of thin films. In this case, ultrathin films floating on water can be caught and connected to a motorized stage and to a load cell on opposite sides and then stretched until failure while measuring both the applied load and displacement in real time.^59^ Although tensile tests of films on water allow researchers to derive complete stress-strain curves, while circumventing typical issues related to the manipulation of completely freestanding specimens as well as limiting the artifacts related to the presence of a solid substrate, concerns yet exist about the possible role played by water on the mechanical behavior shown by the tested films, which can potentially experience some morphological or structural modification as a consequence of the contact with water.^31^

The support offered by water can also be exploited to perform other kinds of mechanical characterization tests. For example, capillary wrinkling tests exploit the fact that when sub-micrometer thin films are afloat on water and are stretched by the surface tension of the air-water interface, they develop a specific wrinkle pattern.^60^ From the number and length of the developed wrinkles, it is then possible to derive properties, such as the Young’s modulus and the thickness of the film under evaluation. Beyond the above discussed techniques, other testing strategies have been reported based on liquid/solid interface, such as elastocapillary bending, which exploits bending of thin films driven by interfacial tension to derive the Young’s modulus of thin films with sub-millimeter in-plane size.^61^

### Mechanical testing of freestanding film

With reference to completely freestanding films, the main testing configurations reported in the literature involve either uniaxial loading, as in tensile tests, or biaxial loading, such as in atomic force microscopy (AFM) deflection test and bulge test. In the case of both AFM deflection test and bulge test, the specimen under testing is a film deposited onto a substrate patterned with holes, typically of circular geometry and with a diameter ranging from a few to hundreds of μm,^62^^,^^63^ in order to provide local access to completely freestanding membranes clamped on their periphery. In AFM deflection tests, the membranes are loaded at the center by the tip of an AFM, which allows us to record the force (F) necessary to progressively deflect the membrane. Force-deflection data can then be curve-fitted according to analytical models derived from continuum mechanics, which allow us to determine mechanical quantities, such as the film’s Young’s modulus and pre-tension. For example, in the case of a clamped circular thin plate, a point force applied at the center can be related to the deflection at the center (*δ*) as^30^

(Equation 2) $$F=\frac{4\pi Et^{3}\delta }{3\left(1-\nu^{2}\right)a^{2}}+\pi \sigma_{0}\delta +\frac{Etq^{3}\delta^{3}}{a^{2}}$$

where *E* is the Young’s modulus, *t* is the film thickness, *a* is the film radius, *σ*_*0*_ is the film pre-tension, and *q= 1/(1.05 – 0.15ν – 0.16ν*^*2*^*)* is a dimensionless constant depending on the Poisson’s ratio, *ν*. Different from AFM deflection test, the bulge test is a noncontact technique yet involves a sample with similar geometry. However, in this case, the film is loaded and deformed because of an applied pressure. In this case, biaxial stress-strain (σ-ε) curves of the film can be built based on the following equations^64^:

(Equation 3) $$\sigma_{11}=\sigma_{22}=\frac{\mathrm{PR}}{2t}$$

(Equation 4) $$\varepsilon_{11}=\varepsilon_{22}=\frac{s}{2R_{0}}-1$$

Where *P* is the applied pressure, *R* is the curvature radius of the membrane, *t* is the membrane thickness, *s* is the segment length of the bubble, and *R*_*0*_ is the hole radius. By monitoring the temporal evolution of the bubble height by an AFM, it is possible to derive information about the creep of the film.

Regarding tensile tests, in the very last few years, some specific experimental setups have been developed. For example, recently Bay & Crosby proposed the tensile tester for ultrathin freestanding films (TUFF),^40^ which allows us to characterize films with mm^2^ area and thickness as small as 30 nm. In this case, the specimen is supported by a metal frame, which is connected to a linear stage in order to apply a tensile load/displacement to the film, whose opposite side is instead attached to a cantilever with calibrated stiffness, enabling force/displacement measurement. A novel tensile testing platform has been developed to characterize freestanding films with thickness of 85–200 nm and a testing area as large as 0.3 mm^2^.^65^^,^^66^ This approach involved the transfer of the film specimen from its native substrate onto a double pre-notched silicon slice, where it lies also during the mechanical test. The pre-notch on topside allows the film to be decoupled from the substrate (i.e., freestanding) over the desired testing region, while being fully supported elsewhere. Before the test starts, by the means of a custom-made clamp, the silicon substrate is fractured in two facing blocks with a tiny gap separation of < 1 μm, which are then attached to a linear stage and to a load cell, respectively. Galuska et al. introduced a testing strategy^31^ where the polymer film (width and gage length of a few mm and thickness down to 19 nm) is initially supported by a water-soluble sacrificial layer on a silicon substrate. Such supported film is connected to a motorized stage, able to deliver controlled, continuous, in-plane shear movement. Upon removing the silicon substrate, the film is attached to a load cell on one side and a linear actuator on the other side and tested.^31^ In addition to macroscopic-sized tensile testing platforms, there are few recent examples reported in the literature of the application of miniaturized tensile testing stages based on the technology of microelectromechanical systems (MEMS).^43^^,^^46^ For example, the nanomechanical device reported by Fang et al.^43^ includes a number of inclined beams able to convert the compression force delivered by a nanoindenter into a tensile load applied to the nanofilm under testing. The compatibility of MEMS-based devices with scanning electron microscope (SEM) enables high-resolution observation of the specimen during the test.

## Mechanical properties of ultrathin polymeric films

**Figure 4:**
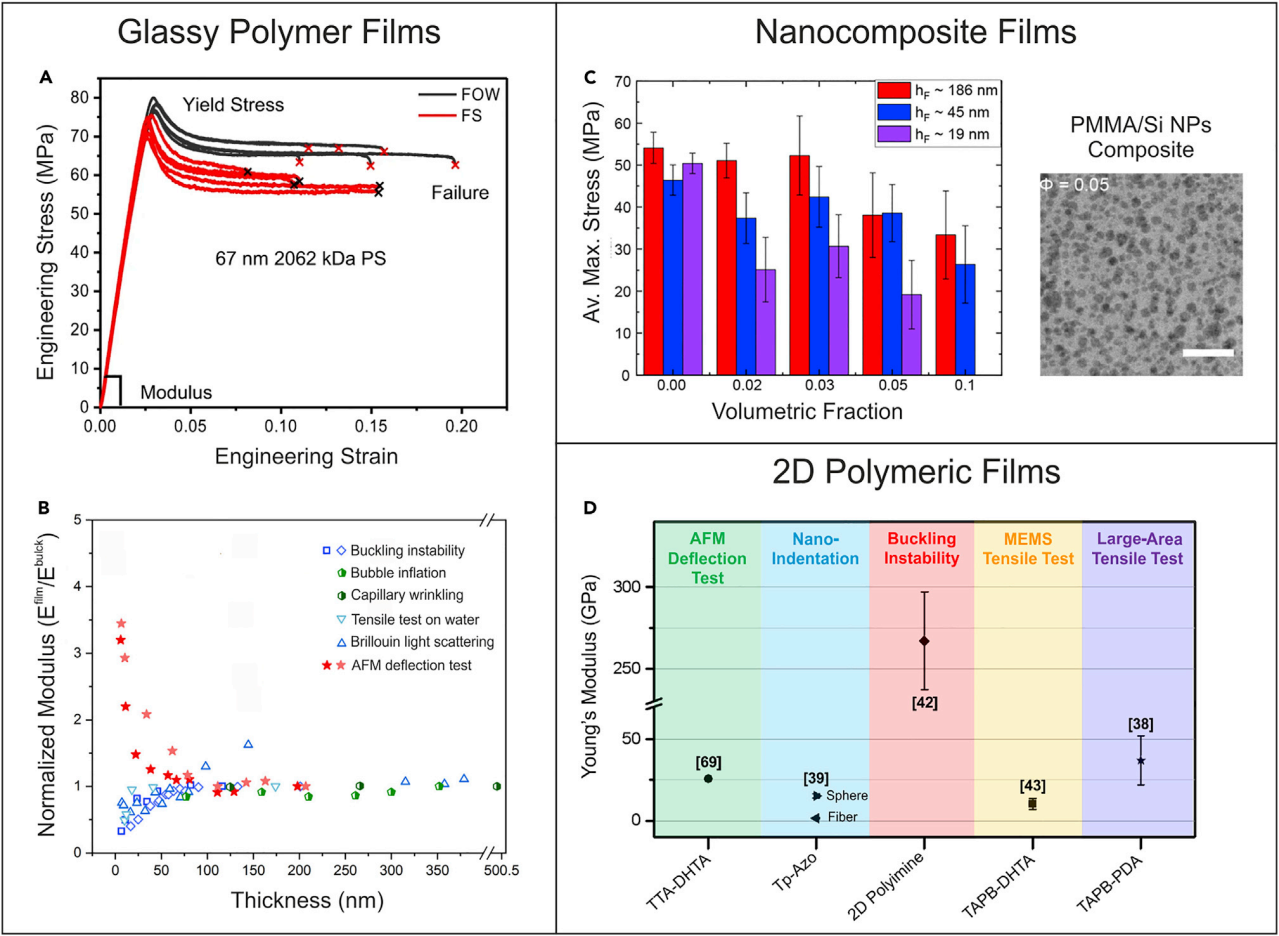
Mechanical properties of ultrathin polymer films (A) Stress-strain curves obtained from tensile tests on PS films of different thickness floating on water. Reprinted under the terms of the Creative Commons CCBY license from Galuska et al.^31^ (B) Young’s modulus of PMMA films as a function of film thickness as determined via different testing techniques. Values are normalized with respect to the Young’s modulus of bulk PMMA. Adapted from Wang et al.^30^ (C) Strength of PMMA films with different thickness reinforced with silica nanoparticles at different particle volume fractions (φ). Reprinted with permission from Bay et al.^68^ (D) Young’s modulus of imine-linked 2D COF nanofilms obtained by different mechanical characterization methods. Adapted from Pantano et al.^38^

In the class of ultrathin polymeric films, glassy polymers are the most widely investigated, and among those, PS and PMMA have emerged as model materials to study the mechanical behavior of polymers in the shape of ultrathin films (Table 1). There are several experimental evidences showing a deviation of the mechanical properties of polymer ultrathin films from their bulk values. For example, tensile tests on water revealed that PS films experience a significant loss in terms of both load standing (from 48 ± 5 MPa for ∼208 nm thin films to 26 ± 1 MPa for ∼21 nm thin films) and deformation capability (strain at failure decreases from ∼8% for films thicker than 150 nm to ∼1.3% for ∼20 nm thin films), when their thickness decreases to 20 nm.^67^ However, the dependence of the mechanical properties on thickness is not always clear, especially if data obtained via different techniques are compared (Figure 4A). Indeed, tensile tests conducted on freestanding PS films with thickness ranging from 32 to 100 nm showed basically constant strength and Young’s modulus, with the latter falling in the bulk range of ∼2.3–3.5 GPa.^40^ Similarly, the application of buckling instability metrology on PS films with thicknesses between 30 and 280 nm revealed an elastic modulus almost constant and equal to 3.4 ± 0.1 GPa,^53^ in good agreement with the results obtained from the tensile tests on freestanding films reported by Galuska et al.^31^ In a later study of buckling instability,^55^ Stafford et al. investigated the mechanical properties of PS films with thickness between 5 and 200 nm, which revealed that PS films with a thickness lower than 30 nm show a decrease in the apparent elastic modulus by up to an order of magnitude if compared to bulk films. As for PS, also data currently available for PMMA show a contradictory variation of the Young’s modulus with thickness^30^ (Figure 4B). For example, bulge tests and BLS tests showed a similar constant trend, while instead AFM deflection tests revealed pronounced stiffening of the films when the thickness is below ∼50 nm. A completely different scenario appears instead when looking at the data obtained from surface wrinkling and tensile testing on water, from which the Young’s modulus was found to decrease with the film thickness.

**Table 1:** Overview of the mechanical properties of selected ultrathin polymeric films evaluated via different techniques

Material	Method	Thickness [nm]	Max stress [MPa]	Young’s modulus [GPa]	Strain at failure (%)	Reference
PS	Tensile test of freestanding film	∼30	∼48.7 ± 8.9	∼2.8 ± 0.3	∼1.5–2.25	Bay et al.^40^
PS	Tensile test of freestanding film	19–155	<50- ∼90	∼3	∼5-22	Galuska et al.^31^
PS	Tensile test of film on water	∼30	∼49.5 ± 9.9	∼3.5 ± 0.5	∼1.75–2	Bay et al.^40^
PS	Buckling instabilities	30–280	–	3.4 ± 0.1	–	Stafford et al.^53^
PS	Brillouin light scattering	40–1,400	–	∼5.5–6	–	Gomopoulos et al.^47^
PS	Capillary wrinkling	6.8–993	–	5.9	–	Chang et al.^49^
PMMA	AFM deflection test	6–200	–	∼9–3	–	Wang et al.^30^
PMMA	Brillouin light scattering	40–500	–	∼7.8	–	Gomopoulos et al.^47^
PMMA	Tensile test of freestanding film	180–280	41 ± 5	2.6 ± 1.1	12 ± 3	Pantano et al.^66^
PMMA	Buckling instability	6.7–116.1	–	∼1–3	–	Stafford et al.^55^
PMMA	Tensile test of freestanding film	19–186	∼45–55	∼3	∼2–2.5	Bay et al.^68^
PMMA	Capillary wrinkling	7.2–545.1	–	4.6	–	Chang et al.^49^

In general, the impact of the thickness reduction on the mechanical properties of thin films depends on many factors, including polymer architecture, backbone stiffness, and additives.^27^ In the case of polymer nanocomposite films (Figure 4C), a recent investigation on composite ultrathin films made of PMMA with embedded silica nanoparticles revealed that the Young’s modulus is poorly affected by the film thickness and nanoparticle loading^68^; on the contrary, the strength of the films showed a thickness-dependent decrease with increasing reinforcement volume fraction. In this case, it is suggested that such decrease in strength can be ascribed to changes in the entanglement network.^68^

Nanofilm membranes composed of polyamide and polyarylate for desalinization and liquid separation applications have been recently prepared via interfacial polymerization by Livingston and co-workers.^2^^,^^11^ The characterization of the polyamide^2^ and polyarylate^11^ nanofilms by bucking instability resulted in a Young’s modulus of 3.57 ± 0.60 GPa and 4.8 GPa, respectively; such values are much higher than the data reported for polyimide membrane prepared by spin coating methods, thus demonstrating once again the importance of the nanofilm preparation route on the mechanical properties. Recently, the mechanical characterization of nanomembranes comprising 2D COF films has been attracting increasing interest.^38^^,^^39^ Figure 4D shows the Young’s modulus of different imine-linked 2D COF films, which are the most investigated among COFs, derived from different testing methods, including AFM deflection test and nanoindentation, buckling instability, and tensile tests. The tested films revealed a large variation in the mechanical properties, with the Young’s modulus ranging from 1.46 ± 0.15 GPa to 267 ± 30 GPa, which can be ascribed not only to the different structure of the imine-linked COFs but also to different preparation strategies and approaches for film transfer and testing methodologies. By using AFM indentation, the Young’s modulus of an imine-linked 2D COF thin film, the TTA-DHTA COF, with thickness of 4.7 nm was determined as 25.9 ± 0.6 GPa.^69^ A recent investigation performed by nanoindentation demonstrated that COF films assembled from nanospheres were much stiffer (15.3 ± 1.28 GPa) compared to COF films obtained from the same monomers but assembled in the form of nanofibers (1.46 ± 0.15 GPa).^39^ Bucking instability was employed for testing a 2D polyimine monolayer deposited on a PDMS support resulting in a Young’s modulus of 267 ± 30 GPa.^42^ Freestanding COF films of similar chemical structure, the TAPB-PDA COF^38^ and the TAPB-DHTA COF,^43^ were recently characterized by means of two different custom-made tensile testing stages. In the first case, a dedicated MEMS-based platform^43^ was applied on ∼50 nm thin TAPB-DHTA COF film. The tested COF films were found to possess a Young’s modulus of 10.38 ± 3.42 GPa, strength of 0.75 ± 0.34 GPa, and critical stress intensity factor of 0.55 ± 0.09 MPa $\sqrt{m}$. In the second case, the TAPB-PDA COF films were instead characterized by a macroscopic tensile testing platform, which revealed a strength of 188 ± 57 MPa and Young’s modulus of 37 ± 15 GPa on a large-area freestanding film. The different mechanical properties observed for the TAPB-DHTA and TAPB-PDA COF films were ascribed to the different synthetic approach and transfer methods used to prepare the COF specimens. Compared to other 2D covalent organic polymers with different linkage chemistries, a 1.7 nm thick sheet obtained from photochemical anthracene [4 + 4]-cycloaddition dimerization was characterized by AFM indentation resulting in a Young’s modulus of about 11 GPa.^70^ Recently, a highly oriented polyaramid 2D polymer, obtained by irreversible polycondensation reaction, showed a Young’s modulus of 12.7 ± 3.8 GPa and a strength of 488 ± 57 MPa.^71^ For comparison, the Young’s modulus of 2D inorganic-organic framework materials, such as 2D metal-organic frameworks (MOF), which are widely used as separation membranes,^15^ was found to be between 3 and 7 GPa.^72^^,^^73^ The higher mechanical performances of 2D COF films, along with their good separation properties,^15^ make COF membranes competitive candidates for nanofiltration and gas separation.

## Nanoconfinement effect on the mechanical properties of ultrathin polymeric films

**Figure 5:**
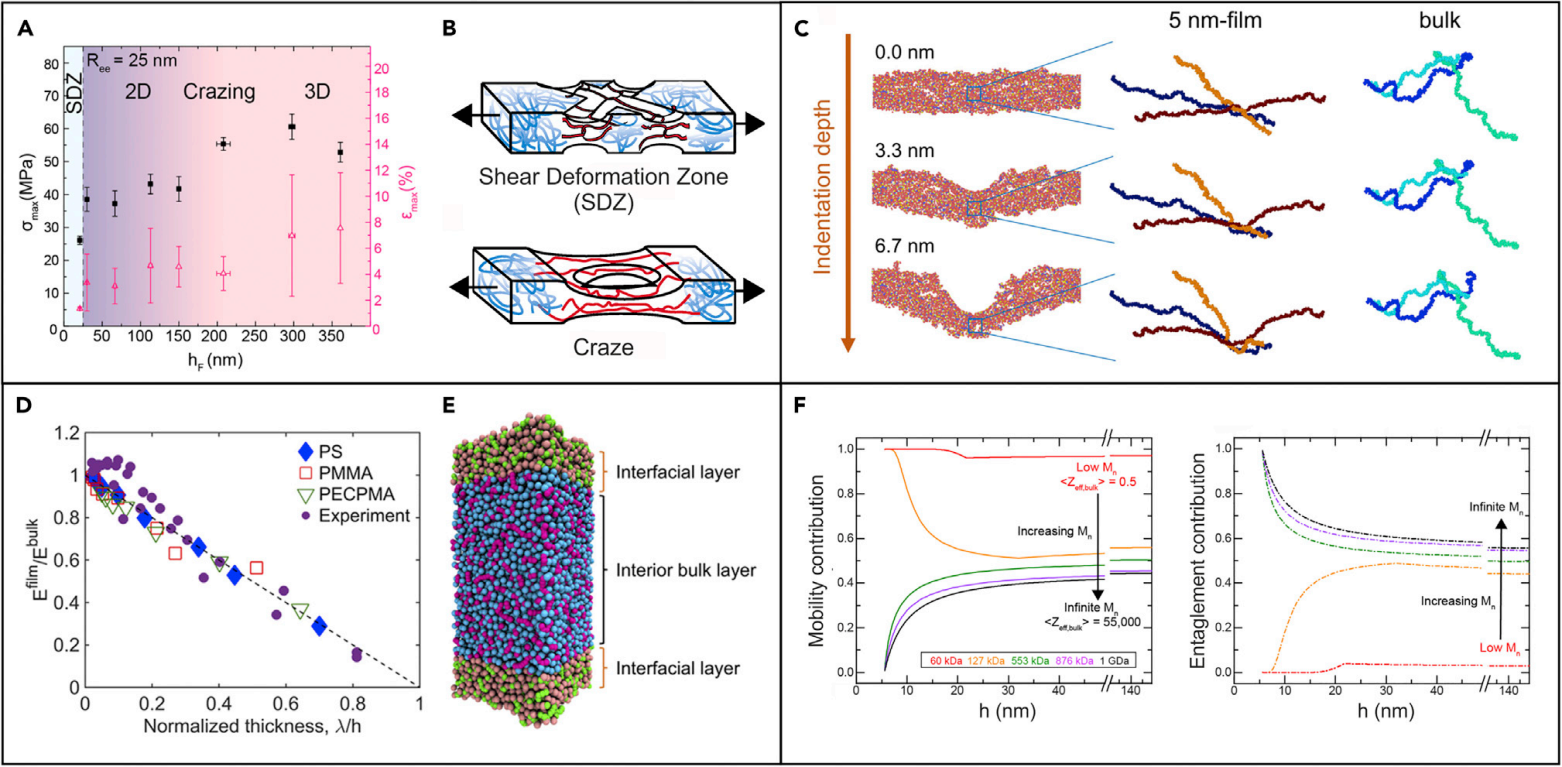
Nanoconfinement effect (A) Strength (left axis) and strain at failure (right axis) of PS films as a function of thickness with highlighted most favorable strain localization mechanism. Reprinted with permission from Bay et al.^67^ (B) Schematic representation of the mechanical behavior of ultrathin films under strain resulting in SDZ or in a craze with a void. Reprinted with permission from Bay et al.^67^ (C) Representation of the chain deformation at different deflections under a tip for a 5 nm thin film and bulk. Polymer chains disentangle and tend to get extended and oriented parallel to the surface upon thickness reduction. Thus, the mechanical response results as governed by chain stiffness. Reprinted with permission from Wang et al.^30^ (D) Thin film Young’s modulus normalized by the corresponding bulk value as a function of the normalized thickness obtained from the CG models (dashed line) of three polymers (PS, PMMA, and PECPMA) and comparison with experimental data (for PS and PMMA films) obtained via buckling metrology. (E) Schematic of the layered composite model, comprising two softer interfacial layers and a stiffer interior bulk layer, developed to evaluate the elastic modulus of freestanding thin films. Reprinted with permission from Xia and Lan.^75^ (F) Impact of chain mobility and interchain entanglements on the yield stress of PS ultrathin films with different molecular weight and thickness. Reprinted with permission from Bay et al.^76^

Understanding the mechanisms underpinning confinement effects is a matter still under debate, which makes an accurate prediction of the mechanical behavior of ultrathin polymeric films still far to be achieved.^74^ In general, when glassy polymers, such as PS and PMMA, are confined to films with thickness approaching a critical size, there are modifications in their average chain mobility (that increases) and structure, including entanglement density; such modifications, in turn, affect the polymer capability to respond to an applied load.^67^ It has been reported that when thickness decreases, polymer chains tend to interact more with themselves than with their neighbors, thus leading to a reduction in the interchain entanglement density. Both chain mobility and interchain entanglement density are known to control strain localization mechanism and can then cause a transition from shear deformation zone (SDZ) to crazing (Figures 5A and 5B). As experimentally observed,^67^ the transition from crazing (for film thickness >30 nm) to SDZ for 20 nm thin films confirmed that the surface mobility is the dominant effect controlling the failure mechanism, as also supported by the observed reduction in the glass transition at smaller thicknesses. However, the reduced interchain entanglement density can then be responsible of an overall embrittlement of the film, with reduced strength, stiffness, and elongation capability at smaller thicknesses.

However, other experimental evidence showed instead an almost constant Young’s modulus even for thicknesses below the end-to-end distance of a polymer chain. Such findings may suggest that more than geometrical constraints, the polymer-surface interaction could play a major role.^31^ In order to achieve more insight into the nanoconfinement effect of ultrathin films as experimentally observed, mechanical tests can be supported by spectroscopic investigations, such as small-angle X-ray scattering (SAXS) experiments, as well as molecular dynamics (MD) simulations. For example, with still reference to PMMA, recent AFM deflection tests showed a pronounced stiffening behavior of freestanding films, when their thickness goes below 2*R*_*g*_, *R*_*g*_ being the radius of gyration that characterizes a random coil conformation.^30^ SAXS measurements revealed that upon thickness reduction the polymer chains disentangle and tend to get extended and oriented parallel to the surface, unlike a bulk polymer. Within the same study, MD simulations unveiled that the conformational transition dominated the deformation of the polymer chains, highlighting the more significant entropic contributions over enthalpic contributions for the chain stiffening mechanism (Figure 5C). By employing an atomistically based coarse-grained (CG) modeling approach, Xia and Lan demonstrated the importance of local molecular stiffness in governing the reduction of the elastic moduli with decreasing film thickness for freestanding PS, PMMA, and poly(1-ethylcyclopentyl methacrylate) (PECPMA) films (Figure 5D).^75^ The local molecular stiffness was evaluated by probing the Debye-Waller factor (DWF), which is related to the segmental “free volume” explored by the polymer chains. This is a critical factor when evaluating the physical properties of nanoconfined polymers and the suppression of segmental dynamics, and therefore the DWF was observed for ultrathin polymer films by incoherent neutron scattering (INS) measurements.^77^ The theoretical approach based on the DWF revealed a softer nanometer-sized surface layer, which allows the development of a layered (composite) model, consisting of two softer free-surface layers and one stiffer bulk-like interior region, to explain the thickness dependence of the elastic modulus of freestanding thin films (Figure 5E).^75^

By using a combination of experiments and simulations, Crosby and co-workers^76^ developed a semiempirical model to understand the structure-property relationships that determine the failure response of glassy polymer films. In order to decouple the effect of the interchain entanglements and chain mobility on the mechanical properties of the films, they first characterized the tensile response of PS films with thickness between 10 and 150 nm and for a range of molecular weights (from 61 to 2,135 kDa), and then they simulated the failure of the films via nonequilibrium MD simulations. For high-molecular-weight polymers, the mobility contribution to the film yield stress decreases while the entanglement contribution increases as the film thickness decreases (Figure 5F). On the contrary, when the number of entanglements per chain decreases, as for low-molecular-weight polymers (127 kDa), the entanglement contribution decreases while the mobility contribution increases as the film thickness decreases. This study demonstrates the different roles played by entanglements and mobility on the failure properties of ultrathin glassy films and that both aspects have to be accurately taken into account in order to design robust films with desired mechanical behavior.

Regarding 2D polymeric films, there is still no experimental evidence of the dependence of the mechanical properties on the thickness of the film, in particular for few-layer membranes, because the evaluation of the mechanical properties of such nanometer-thin 2D films remains an undeniable challenge.

## Challenges and limitations

As evident from the literature analysis, it is challenging to create a coherent theoretical framework explaining the mechanical behavior of ultrathin polymer films. Such difficulties stem from the often-contradictory evidences found during experiments, which originate from the adoption of a wealth of different testing techniques involving different loading conditions and specimen preparation. In turn, the lack of universal methods and protocols comes from the difficulties related to the manipulation of films with nanoscale thickness, which, with respect to standard tensile tests, has attracted the use of mechanical characterization techniques involving films deposited onto a substrate, such as nanoindentation, BLS, or buckling metrology. However, for example, in the case of nanoindentation, the peculiar loading condition involved during the measurement makes the mechanical response very sensitive to the nanoindenter tip and the presence of the underlying substrate, with the possibility to introduce artifacts that increases when the thickness of the film decreases. Such artifacts can in the end overlap with the effects due to nanoconfinement, which may then be difficult to isolate. In order to minimize substrate effects and improve the reliability of the obtained results, testing parameters (e.g., choice of the substrate and indentation depth) have therefore to be carefully chosen.^78^ On the other hand, those techniques are effective for testing thin films deposited on devices, as protective coating of the device itself or as electrical and optical coatings. As an alternative option, noncontact methods, like BLS or buckling metrology can be considered.^79^ BLS has the interesting capability to determine either in-plane or out-of-plane elastic modulus of thin films, depending on the direction of the collected backscattered light,^47^ yet it requires specific setup, including a laser source, and poses questions about the suitability to derive the elastic modulus of films with <40 nm thickness.^27^ Buckling metrology is a very popular technique, which has been broadly used for a variety of thin films. It offers the possibility to test simultaneously large-area samples with even extremely low thickness (Table 1), and its resolution can be basically tuned and improved according to the strategy adopted for the measurement of the wrinkle pattern wavelength. The evaluation of the Young’s modulus by this technique, however, relies on a number of assumptions.

In order to solve issues related to the substrate effect, it would be ideal to test a specimen that is freestanding. Techniques, such as AFM deflection test or the bulge test, for example, take advantage of a relatively straightforward sample preparation and address the issue related to the manipulation of completely freestanding nanoscale thin films, as these are prepared as deposited onto a substrate with pre-patterned holes. In this kind of tests, the stress field applied to the specimen is essentially biaxial, and the derivation of mechanical properties, like the Young’s modulus, can be severely affected by the tip geometry and contact (for the AFM test) and rely on analytical models derived from continuum mechanics, which have to obey to a number of assumptions, as well.

The above mentioned techniques allow us to infer about the mechanical behavior of the tested samples mostly limited to the Young’s modulus. On the contrary, tensile tests allow us to derive the full stress-strain curve of the samples, from which a wealth of data can then be extracted, including the elastic modulus, strength, strain at failure, and eventual yielding behavior. In general, with respect to testing supported films, tensile tests of completely freestanding films require additional efforts in sample preparation and handling, which up to now have limited the possibility to test films with <30 nm thickness, with just a few reported exceptions, such as the recent tensile tests on PS films with 19 nm thickness enabled by SMART transfer.^31^

## Current directions and future perspectives

In the future, the success of ultrathin polymeric film-based devices will strictly depend on the capability of ultrathin polymeric films to adequately respond to the wide variety of mechanical demands of real operating conditions. This represents a critical question to be addressed, and still, today it is difficult to provide an exhaustive answer.

Below we discuss promising topics that, if resolved, will allow us to derive the mechanical properties of thin films and understand whether they are suitable to support a certain application.

i) For practical applications of thin films, more straightforward and high-throughput techniques are needed for their mechanical characterization. This is of fundamental importance in order to provide a deep understanding of the mechanical properties of libraries of thin film materials, whose preparation has been recently accelerated by automated synthesis platforms,^80^ before their actual implementation in the device;
ii) Most of the studies reported up to now were mainly focused on quasi-static properties, while dynamic behavior, fatigue, creep, or viscoelastic relaxation (that can be severe phenomena in the case of polymers) have been scarcely investigated. On the contrary, their investigation is important to enable full functionalities of ultrathin polymeric film devices, especially for high-rate, high-frequency, or long-life applications;
iii) It will be exciting to understand the structure-mechanical properties relationship of thin polymeric films. Devising accurately the mechanical properties of films with different composition and structure is critical in order to integrate the experimental data with simulations for enhancing the reliability and predictability of the mechanical properties of thin films.

In summary, the emergence of novel fabrication approaches and organic materials in recent years has accelerated progress in the design of advanced, high-performance ultrathin membranes for a wide range of engineering applications, such as ultrafiltration membranes and wearable devices. However, despite their tremendous potential, practically, ultrathin membranes still pose significant challenges, with questions about their robustness and stability over broad application conditions, and with a deep understanding of their mechanical properties being yet to be achieved; future research has to address such challenges by pursuing a more systematic experimental work on the mechanics of polymeric thin films to enable capturing those mechanisms and features, which, if properly understood and mastered, will ultimately provide the key to design their mechanical behavior on demand.
